# Motivational interviewing retention counseling and adherence to early infant diagnostic HIV testing schedule in South Africa: The PAEDLINK randomized trial: Erratum

**DOI:** 10.1097/MD.0000000000029194

**Published:** 2022-04-01

In the article, “Motivational interviewing retention counseling and adherence to early infant diagnostic HIV testing schedule in South Africa: The PAEDLINK randomized trial”,^[1]^ which published in Volume 101, Issue 6 of *Medicine*, the percentage for groups b and c were written incorrectly in the abstract. Group B's percentage originally appeared as 68.3% and has been corrected to 48.1%. Group C's percentage appeared as 75.3% and has been corrected to 52.7%. This information was correct throughout the rest of the article and does not effect the conclusions.
